# A 37 mm Spindle Cell Lipoma on the Floor of the Mouth

**DOI:** 10.1155/2019/2138928

**Published:** 2019-08-04

**Authors:** Eve Malthiery, Valérie Costes-Martineau, Marie-Alix Fauroux, Jacques-Henri Torres

**Affiliations:** ^1^Service d'Odontologie, Centre Hospitalier Universitaire, 34295 Montpellier Cedex 5, France; ^2^Laboratoire d'Anatomie et de Cytologie Pathologiques, Centre Hospitalier Universitaire, 34295 Montpellier Cedex 5, France

## Abstract

Spindle cell lipoma (SCL) is a rare variety of lipoma that mostly arises in male patients and rarely affects the oral cavity. The floor of the mouth is an uncommon site for SCL, and very few cases have been reported in this location. A case of SCL is reported in a 70-year-old woman who had noticed a swelling of the floor of the mouth without any functional consequence. Both ultrasonography and RMI suggested a diagnosis of ranula, whereas clinical palpation showed a nonfluctuant mass. The lesion was excised under local anesthesia. A 37 × 32 mm encapsulated yellow mass was removed. Histological features (mature adipocytes and CD34+ spindle cells) led to a diagnosis of SCL. Medical imaging assessment of this lesion could have been influenced by the high frequency of the ranulas in the floor of the mouth. This case appears to be quite infrequent because of its location (floor of the mouth), its size (over 3.5 cm), and the patient's gender (female).

## 1. Introduction

Lipomas are not frequent in the oral cavity. They only represent 0.1 to 5% of oral tumors [1]. They usually appear as soft yellowish nodular swellings covered by normal mucosa. Lipomas seldom affect the floor of the mouth (2 among the 46 lipomas reported by Fregnani et al., while 10 were in the buccal mucosa) [1].

Several histopathological types of lipomas have been described: fibrolipoma, sialolipoma, myelolipoma, osteolipoma, chondrolipoma, chondroid lipoma, angiolipoma, angiomyolipoma, myolipoma, intramuscular lipoma, pleomorphic lipoma, and spindle cell lipoma (SCL) [2]. Enzinger and Harvey first described SCL in 1975 [3]. These tumors typically arise in the subcutaneous tissue of the upper back, posterior neck, and shoulders of males aged 40–70 and scarcely involve the oral cavity [4].

In 2003, Fregnani et al. reported 46 cases of lipomas of the oral cavity [1]. Five of them were in the floor of the mouth, none of which were SCL. Although SCL was predominant in the lip in the Furlong et al. 2004 report of 95 lipomas of the oral region, only 1 SCL was detected among the 5 cases reported in the floor of the mouth [5]. In 2006, Billings et al. found 16 published SCL cases of the oral cavity in the literature [4]. They reported 7 new cases of SCL, of which only 1 was located in the floor of the mouth. In their 2010 series of 41 cases of lipomas, Juliasse et al. reported 1 SCL among 3 cases of lipomas in the floor of the mouth [2]. In 2013, Manor et al. found 33 reported cases of SCL of the oral cavity and added 2 personal cases [6]. The average size of these lesions was 20.87 mm; male predilection was 2 : 1. Among these 35 cases, only 5 affected the floor of the mouth.

We are reporting a new case of SCL in the floor of the mouth in a 70-year-old woman.

## 2. Observation

**Figure 1:**
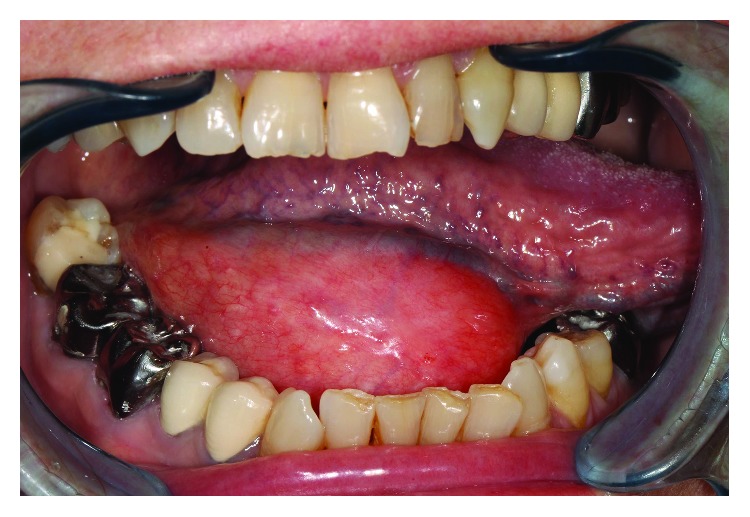
Clinical aspect of the mass located in the right sublingual region (tip of the tongue turned to the left side). The superior limit is above the occlusal surface of the molars.

A 70-year-old Caucasian woman was referred to the hospital by her dentist for a swelling noticed 3 weeks before in the floor of the mouth. It had no functional consequence on her speech or mastication and neither on tongue mobility. An echography was performed, which lead to the conclusion of a ranula. The patient's medical conditions of osteoporosis, hypothyroidism, and mild depression were treated by alendronic acid, levothyroxine, and fluoxetine. Physical examination revealed a tender mass, in the right sublingual region, though its posterior limit could not be clearly defined (Figure 1).

The swelling was not attached to the mandible bone or to other surrounding tissues. The mucosa covering the mass had a normal appearance and color. No inflammation, erosion, or ulceration were noted. Salivary flow from the right submandibular gland was not affected by the swelling. Palpation revealed a nonfluctuant mass without cervical lymphadenopathy.

**Figure 2:**
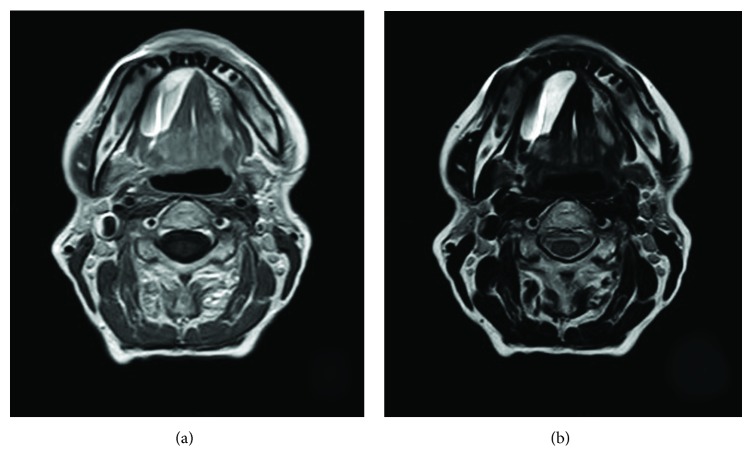
RMI features of the lesion: both T1- (a) and T2- (b) weighed images show hypersignal.

An MRI scan was prescribed, which showed a 37 mm well-defined lesion with both T1 and T2 hypersignals (Figure 2); the conclusion was that the sublingual mass could be a ranula. The patient was proposed an excision, and the procedure was performed under local anesthesia.

**Figure 3:**
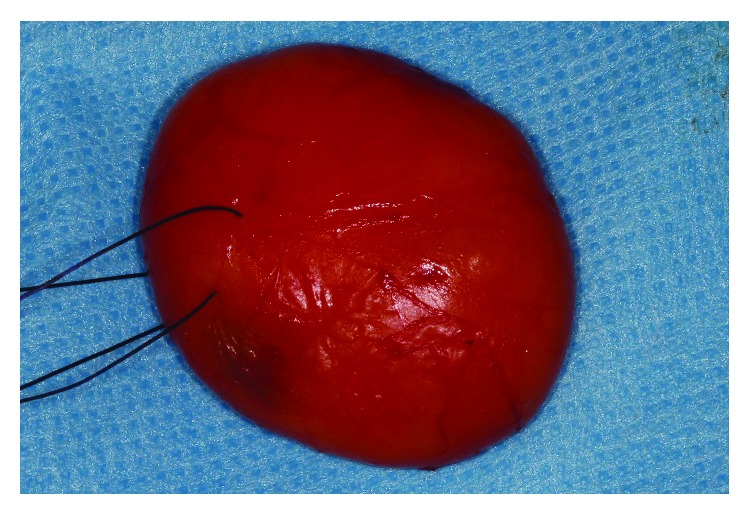
Resection specimen: 37 × 32 mm encapsulated yellow mass.

An encapsulated yellow mass was removed (Figure 3).

**Figure 4:**
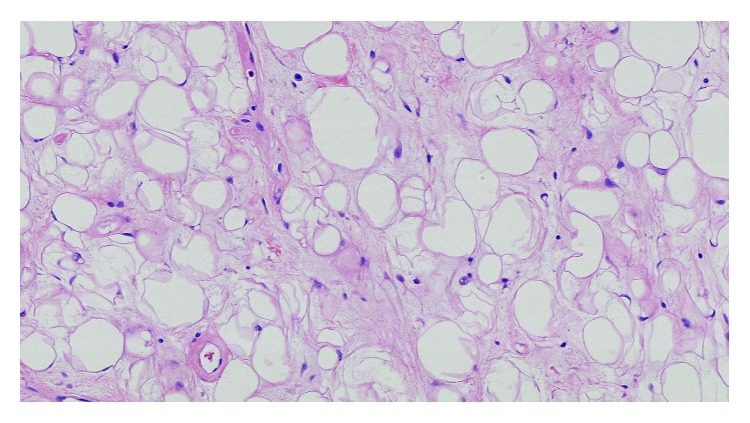
Histology showing mature adipocytes, uniform spindle cells, and short bundles of collagen in a myxoid matrix (HE staining, ×20).

Histopathological examination established a diagnosis of spindle cell lipoma (Figure 4).

**Figure 5:**
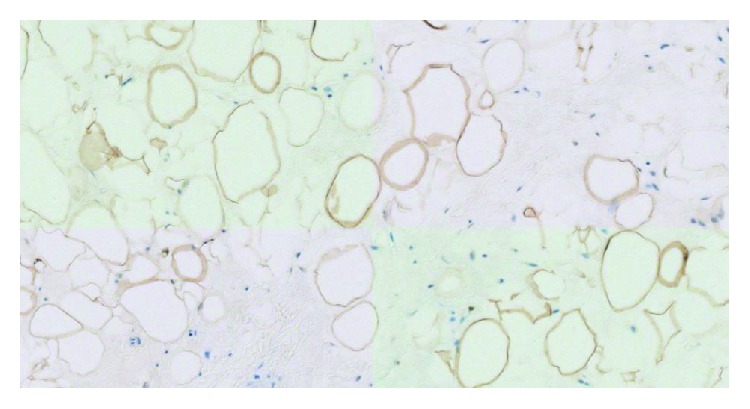
Immunohistochemical anti-PS100 staining (×20): adipocytes are PS100 positive.

**Figure 6:**
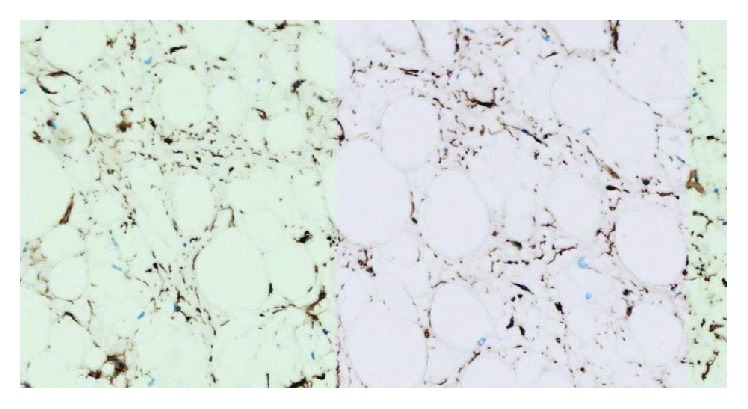
Immunohistochemical anti-CD34 staining (×20): adipocytes are CD34 negative and spindle cells are CD34 positive.

A proliferation of adipocytes in a myxoid background was seen, associated to small bland spindle cells with small chromatic nuclei without mitosis or atypia, low cellular density, and without wavy collagen fibers. Adipocytes size was regular, without significant anisocytosis unlike the recently described anisometric cell lipoma [7]. Immunohistochemical examination was HMGA2 and MDM2 negative. Spindle cells were immunoreactive for CD34 and negative for PS100 (Figures 5 and 6).

## 3. Discussion

Like in the case reported here, oral lipomas are usually asymptomatic. However, large lipomas have been reported to cause functional impairment: a lipoma measuring 5 cm disturbed lower denture stability [8]. An 8.5 cm sublingual lipoma has been reported to cause submandibular salivary gland swelling [9].

In the case series reported by Manor et al. [6], only one SCL in the floor of the mouth was larger (45 mm) than the one reported here (37 mm). Despite the size of the lesion, local anesthesia was chosen for surgery in this case because of patient's cooperation and preference to avoid general anesthesia.

In this case, ultrasonography could not eliminate a diagnosis of the ranula. Yet, a ranula typically appears as an anechoic or hypoechoic cystic component, whereas a lipoma has an isoechoic appearance with multiple internal fine echogenic lines [10]. Clinical palpation was more efficient, showing that the mass was not liquid. At this stage, the most likely clinical diagnosis was a sublingual gland pleomorphic adenoma. On RMI, imaging features of such lesions are typically T1 low signal and T2 marked hyperintensity [11]. The RMI exam, which has not been performed in the hospital, showed T1 and T2 hypersignals. However, the RMI report proposed a diagnosis of ranula. It may also be supposed that the location of the mass influenced the assessment; a ranula is indeed much more frequent than an adenoma or a lipoma of the floor of the mouth. Both lipoma and ranula are supposed to give homogenous internal structure, but lipoma T1-T2 images are expected to be high/high whereas ranula T1-T2 images are supposed to be low/markedly high [12]. In the present case, the RMI appearance was in favor of a lipoma.

From a histologic point of view, SCLs are made up of mature adipocytes and CD34+ spindle cells in a myxoid matrix with bands of birefringent collagen. Their histological differential diagnosis includes benign lesions such as classic lipoma, schwannoma, neurofibroma, leiomyoma, solitary fibrous tumor (whose prognosis is regarded as uncertain) and truly malignant ones such as atypical lipomatous tumors (well-differentiated liposarcoma), and dermatofibrosarcoma protuberans [13]. In particular, secondary degenerative modifications and atrophy observed in SCL must not be mistaken with the malignant histologic features seen in liposarcomas [14].

## 4. Conclusion

This case appears to be very infrequent because of its location (floor of the mouth), its size (over 3.5 cm), and the patient's gender (female). It also reminds the clinicians to prudently consider data from imaging interpretation of tumors.
